# Differential absolute plasma IL-6 concentrations between two immunoassay platforms in intensive care unit patients with COVID-19

**DOI:** 10.2217/bmm-2023-0073

**Published:** 2023-08-31

**Authors:** Pavan K Bhatraju, Eric D Morrell, Nicholas G O'Connor, Audrey Choi, Michael Fitzpatrick, Craig H Smith, Mark M Wurfel, Wayne Conrad Liles

**Affiliations:** 1 Division of Pulmonary, Critical Care and Sleep Medicine, Department of Medicine, University of Washington, Seattle, 98104 WA, USA; 2 Sepsis Center of Research Excellence – University of Washington (SCORE-UW), Seattle, 98104 WA, USA; 3 Kidney Research Institute, Division of Nephrology, Department of Medicine, University of Washington, Seattle, 98104 WA, USA; 4 Department of Medicine, University of Washington, Seattle, 98195 WA, USA

**Keywords:** COVID-19, IL-6, Meso Scale Discovery, Roche, sepsis

## Abstract

**Objectives:**

Explore whether plasma IL-6 levels are similar across biomarker platforms and association with COVID-19 clinical outcomes.

**Methods:**

Plasma IL-6 concentrations were measured on 191 COVID-19 patients using the Roche Elecsys IL-6 assay and the Meso Scale Discovery assay.

**Results:**

Correlation of IL-6 levels between platforms was high (r = 0.87; 95% CI: 0.82–0.89); however, agreement was low (bias: 147.2 pg/ml; 95% limits of agreement: -489.5–783.9 pg/ml). The optimal IL-6 threshold to predict invasive mechanical ventilation and in-hospital mortality were 3- and 3.4-fold higher in Roche compared with Meso Scale Discovery, respectively.

**Conclusion:**

The absolute IL-6 threshold to predict outcomes was consistently higher using the Roche platform, and IL-6 thresholds to inform prognosis vary based on the biomarker platform.

## Tweetable abstract

IL-6 levels in COVID-19 patients are correlated between two different biomarker platforms but the absolute IL-6 levels were consistently higher in samples measured using Roche versus the Meso Scale Discovery device.

A growing body of evidence suggests that plasma IL-6 levels are associated with disease severity in COVID-19 [1]. However, studies investigating IL-6 receptor inhibition to improve clinical outcomes in COVID-19 have been inconsistent. While some studies have demonstrated reduced mortality with tocilizumab (IL-6 receptor inhibitor) [2], others have shown no benefit or even excess mortality [3,4]. These mixed results leave clinicians with uncertainty of which patients may benefit from tocilizumab therapy. In autoimmune diseases, numerous studies have been conducted with the goal of establishing IL-6 thresholds, which can be used to reliably predict treatment response to tocilizumab [5]. In COVID-19, a retrospective analysis of tocilizumab therapy in relation to baseline IL-6 levels showed a significant reduction in mortality (from 36% to 16%) in patients with baseline IL-6 levels above 30 pg/ml but no reduction in mortality in IL-6 below 30 pg/ml [6]. However, to implement such an approach will first require an understanding of the variance in IL-6 measurements across different clinical and research biomarker platforms.

Currently, Roche's Elecsys' IL-6 test has been approved by the US FDA for emergency use authorization in COVID-19 [7]. In addition, a number of research studies have used the Meso Scale Discovery (MSD) biomarker platform to measure IL-6 levels and identify thresholds to predict clinical outcomes in COVID-19 [8,9]. In this study, we investigated whether IL-6 levels are similar across different biomarker platforms and whether clinicians can use one threshold to identify patients at high risk of poor clinical outcomes.

## Materials & methods

### Study design & data source

We prospectively enrolled 191 patients aged ≥18 years admitted to an intensive care unit (ICU) from two University of Washington hospitals with positive reverse-transcription PCR nasopharyngeal swab assay for SARS-CoV-2. Patients were enrolled from 2 April 2020 to 14 May 2021. Plasma was collected using ethylenediaminetetraacetic acid tubes within 24 h of ICU admission. Clinical data were abstracted from the electronic medical record into standardized case report forms by research coordinators. All patients included in this analysis had complete hospital data. The University of Washington institutional review board approved all studies (STUDY9763). Additional details of study enrollment have been previously published [8].

### Plasma IL-6 measurements

The Roche Elecsys IL-6 assay is a chemiluminescent immunoassay conducted on the Cobas e411 immunoassay analyzer (IN, USA). The Roche Elecsys IL-6 assay has a measuring range of 1.5–5000 pg/ml, a limit of quantitation of 2.5 pg/ml, an interassay precision (coefficient of variation) of 17.4% (at 1.82 pg/ml) and 2.0% (at 4461 pg/ml). The stated reference interval is <7 pg/ml [7]. The MSD assay uses patterned arrays and an electrochemiluminescence detection method, which is quantified using the MSD Quickplex SQ 120 instrument (MD, USA). All personnel measuring the biomarkers were blinded to clinical outcomes. Biomarkers were measured for research purposes. All biomarkers were measured with the same number of sample freeze–thaws.

### Statistical analysis

Continuous and categorical variables are reported as median (interquartile range [IQR]) and n (%), respectively. Pearson's correlation and a Bland–Altman plot were generated to compare Roche and MSD IL-6 levels. Next, we constructed area under the receiver operating characteristics curve (AUC-ROC) with a bootstrapped 95% CI and tested against the null hypothesis for the prediction of IL-6 levels and clinical outcomes. We compared differences in the AUC-ROC using Delong's test of equality. We estimated the optimal point on the AUC-ROC for invasive mechanical ventilation (IMV) and in-hospital mortality using the Youden's index, which maximizes sensitivity and specificity. We compared the optimal threshold to identify clinical outcomes between the two IL-6 biomarker assay platforms.

## Results

Among 191 COVID-19 ICU patients, mean (± standard deviation) age was 54.3 (±15.9), and 65% of patients were male (Table 1). Mean (± standard deviation) APACHE (acute physiology and chronic health evaluation) II score was 23 (±9.1). One hundred and thirty-two patients (69%) received corticosteroids and only seven (4%) patients received tocilizumab. Eighty-five (45%) patients received IMV on study enrollment and 70 (37%) died during their hospitalization.

**Table 1.** Patient characteristics among intensive care unit patients with COVID-19.

Variables	COVID-19 (n = 191)
**Demographics**	
Age, mean ± SD	54.3 ± 15.9
Male, n (%)	124 (65)
Race, n (%)	
– American–Indian	8 (4)
– Asian	25 (13)
– Black/African–American	28 (15)
– Pacific Islander	4 (2)
– White	112 (59%)
– Unknown	14 (7)
Ethnicity, n (%)	
– Hispanic/Latinx	63 (33)
Coexisting disease, n (%)	
– Asthma	29 (15)
– Chronic kidney disease	38 (20)
– Coronary artery disease	19 (10)
– Diabetes mellitus	66 (33)
– Hypertension	98 (51)
**Characteristics upon intensive care unit admission**	
– Invasive mechanical ventilation	85 (45)
– Extracorporeal membrane oxygenation	16 (8)
APACHE II, mean ± SD	23 ± 9.1
**Treatments**	
– Corticosteroids	132 (69)
– Tocilizumab	7 (4)
**Outcomes**	
– In-hospital mortality	70 (37)

Data are n (%) or mean (SD). Treatments include receipt of corticosteroids or tocilizumab during hospitalization.

APACHE: Acute physiology and chronic health evaluation; SD: Standard deviation.

**Figure 1.**
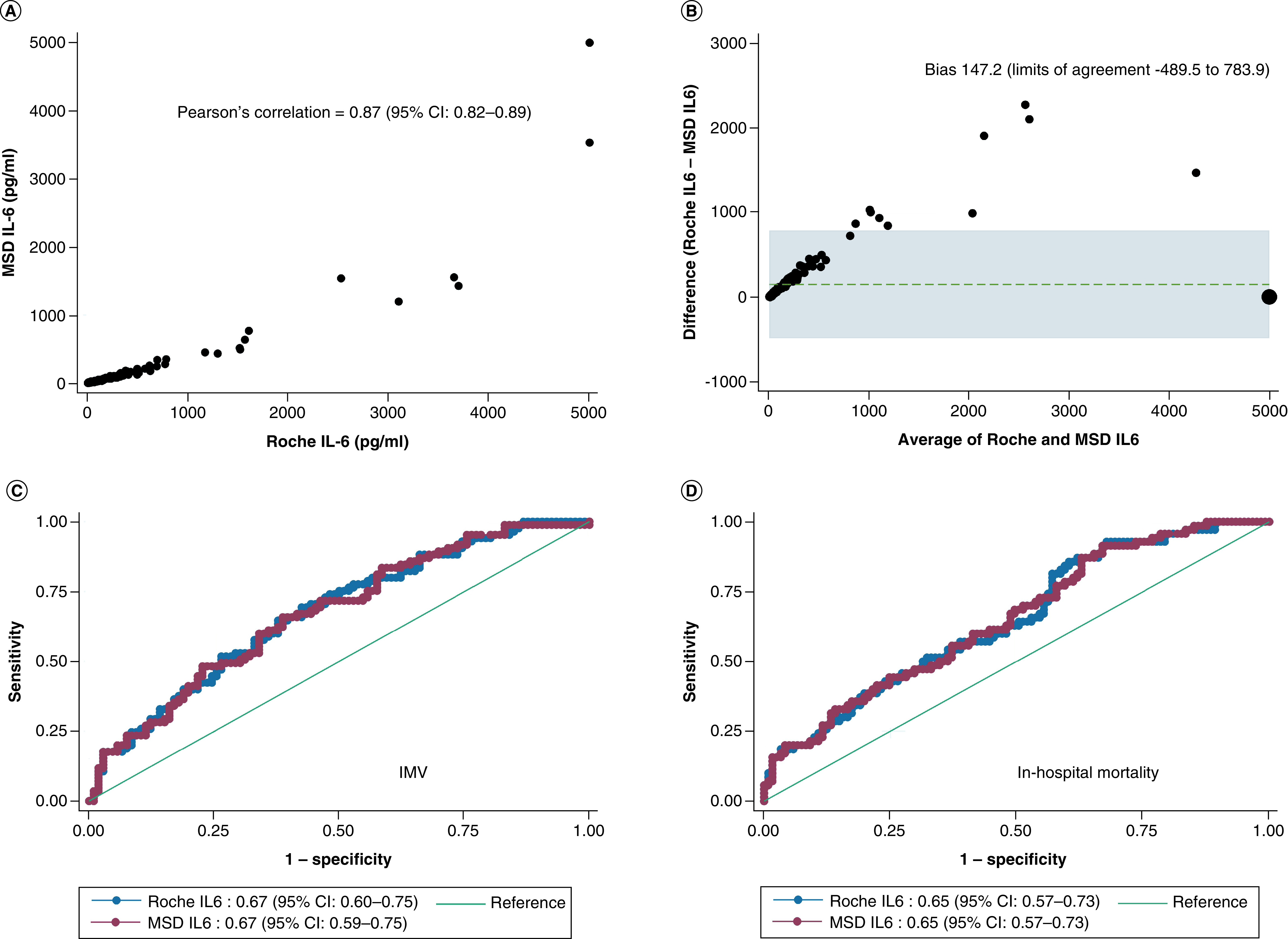
Comparison of IL-6 Concentrations. **(A)** Correlation between MSD and Roche IL-6 levels. p-value for correlation was <0.001. Four patients had MSD values that were truncated at 5000 pg/ml as this was the upper limit of detection for IL-6 on the Roche assay. **(B)** Bland–Altman plot demonstrates poor agreement between Roche and MSD IL6 measurements. Four patients had MSD values that we truncated at 5000 pg/ml as this was the upper limit of detection for IL-6 on the Roche assay. **(C)** AUC-ROC for receiving IMV on study enrollment. The AUC-ROC for Roche IL-6 was 0.67 (95% CI: 0.60–0.75) and for MSD IL-6 was 0.67 (0.59–0.75). These two models were not significantly different (p = 0.79). The Youden's index was 62.1 pg/ml for Roche IL-6 levels which resulted in a sensitivity of 65% and specificity of 62%. The Youden's index was 21.4 pg/ml for MSD IL-6 levels with a sensitivity of 66% and specificity of 61%. **(D)** AUC-ROC for in-hospital mortality for Roche IL-6 was 0.65 (95% CI: 0.57–0.73) and for MSD IL-6 was 0.65 (0.57–0.73). These two models were not significantly different (p = 0.77). The Youden's index for Roche IL-6 was 31.32 pg/ml with a sensitivity of 86% and specificity of 40%. The Youden's index for MSD IL-6 was 9.3 pg/ml with a sensitivity of 91% and specificity of 33%. AUC-ROC: Area under the receiver operator characteristics curve; IMV: Invasive mechanical ventilation; MSD: Meso Scale Discovery.

Roche IL-6 levels had a median (IQR) of 62.0 pg/ml (24.7–224.6) and range of 1.8–5000 pg/ml. MSD IL-6 levels had a median (IQR) of 22.0 pg/ml (9.3–74.7) and range of 0.78–10,387 pg/ml. The coefficient of variation was 9.5% for Roche and 12.3% for MSD. Roche and MSD IL-6 levels were strongly correlated (Pearson's correlation = 0.87; 95% CI: 0.82–0.89) (Figure 1A). However, agreement between assays was low. A Bland–Altman plot demonstrated consistently higher IL-6 levels in samples measured using Roche compared with MSD (bias: 147.2; 95% limits of agreement: -489.5 to 783.9) with a greater variance with higher IL-6 concentrations (Figure 1B). In addition, we also enrolled a comparator population of non-COVID-19 patients admitted to the ICU (n = 106) and measured IL-6 levels using the Roche Elecsys assay. In the non-COVID-19 patients, the Roche IL-6 levels had a median (IQR) of 68 pg/ml (25.8–346.7). The Roche IL-6 levels were overlapping between COVID and non-COVID-19 patients.

To determine whether the difference in IL-6 levels between biomarker platforms was due to differences in sample handling or differences in the calibration of each platform, we ran control standards with a known concentration of IL-6 on the Roche machine. This experiment replicated the bias demonstrated in patient samples and verified that Roche IL-6 measurements were higher than MSD IL-6 levels. For example, standard 5 had an average MSD IL-6 value of 2.94 pg/ml and an average Roche IL-6 level of 8.52 pg/ml or a 2.89-fold higher difference. Standard 1 had a MSD IL-6 level of 798 pg/ml and a Roche IL-6 value of 1689 pg/ml or 2.12-fold higher difference.

Both Roche and MSD IL-6 levels were predictive of IMV on study enrollment. Roche IL-6 levels had an AUC-ROC of 0.67 (95% CI: 0.60–0.75; p < 0.001) and MSD IL-6 levels had an AUC-ROC of 0.67 (95% CI: 0.59–0.75; p < 0.001) (Figure 1C). Delong's test found that these two models were not significantly different (p = 0.79). We also found that both Roche and MSD IL-6 levels on study enrollment were predictive of in-hospital mortality and found these two models were not significantly different (p = 0.77) (Figure 1D). In a sensitivity analysis, we tested whether IL-6 levels were associated with hospital mortality in patients receiving IMV on study enrollment. Among 191 patients, 85 were receiving IMV at study enrollment and 36 (42%) subsequently died during hospitalization. In patients receiving IMV who survived hospitalization, Roche IL-6 level was a median (IQR) of 81 pg/ml (34–264) and in patients who died it was 160 pg/ml (50–1149) with a statistically significant difference in IL-6 levels (p = 0.021).

The optimal IL-6 threshold to predict IMV was three-times greater and to predict in-hospital mortality was 3.4-times greater for Roche than MSD. The optimal IL-6 threshold for Roche was 62.1 pg/ml with a sensitivity of 65% and specificity of 62% for prediction of IMV. In contrast, the optimal IL-6 threshold for MSD was 21.4 pg/ml with a sensitivity of 66% and specificity of 61%. The optimal IL-6 threshold for predicting in-hospital mortality for Roche was 31.3 pg/ml with a sensitivity of 86% and specificity of 40% and the optimal threshold for MSD was 9.3 pg/ml with a sensitivity of 91% and specificity of 33%.

Next, we tested the performance of the Roche IL-6 thresholds for IMV and in-hospital mortality to risk stratify patients using MSD IL-6 values and calculated the cross-tabulation of ‘high’ and ‘low’ IL-6 patient groups. Overall, we found poor overlap of patient assignment from Roche to MSD IL-6 levels. Using the Roche IL-6 threshold of 62.1 pg/ml for IMV identified 95 patients with high IL-6 levels and 96 with low. However, using this same threshold for MSD identified only 54 patients with high IL-6 levels and 137 patients with low, giving a moderate Cohen's kappa for group agreement of 0.57. Similarly, the optimal Roche IL-6 threshold of 31.3 pg/ml for in-hospital mortality identified 134 patients with high Roche IL-6 values and 57 patients with low values. However, using this same threshold for MSD IL-6 identified 111 patients with high IL-6 levels and 80 patients, yielding a Cohen's kappa for group agreement of 0.47.

## Discussion

To our knowledge, this is the largest study directly comparing IL-6 values measured using two different biomarker platforms in the same set of ICU patients with COVID-19 [10]. Although IL-6 concentrations are highly correlated between the Roche and MSD biomarker platforms, agreement between values is low. Overall, Roche IL-6 values were two- to threefold higher than MSD values. Both Roche and MSD IL-6 levels were predictive of IMV and in-hospital mortality; however, optimal Roche IL-6 thresholds to identify patients at high risk of poor clinical outcomes did not identify the same patients using MSD IL-6 values. These findings have multiple clinical implications, including generalizability of IL-6 biomarker measurements across different studies and defining optimal thresholds for clinical treatment decisions. Data from alternative studies have shown the prognostic importance of IL-6 levels in COVID-19 but a direct comparison between the MSD and Roche assays are lacking. Previous studies have found that MSD has the widest dynamic range compared with other immunoassays for measuring IL-6 levels [11] and that the Roche IL-6 assay is associated with clinical outcomes in COVID-19 [7].

Our findings highlight the challenges with comparing absolute IL-6 values across multiple studies using different biomarker platforms. Early in the pandemic, a number of retrospective analyses compared IL-6 levels between patients with COVID-19 ARDS (acute respiratory distress syndrome) compared with non-COVID-19 ARDS or non-COVID-19 sepsis. However, these analyses combined IL-6 values measured using multiple different biomarker platforms and this limits comparison of absolute biomarker levels. Moreover, identification of absolute thresholds of IL-6 levels that are associated with clinical outcomes can inform identification of patients for therapeutics that block the IL-6 pathway, such as tocilizumab. Tocilizumab currently has a moderate recommendation by the NIH COVID-19 therapeutic guidelines for use in hospitalized patients that are rapidly worsening despite initiation of dexamethasone therapy. We suggest that future analyses comparing absolute IL-6 levels should complete measurements using the same biomarker platform or consider adjustment factors for standardization.

## Conclusion

IL-6 values have poor agreement between an FDA-approved Roche IL-6 assay and an MSD IL-6 assay that is commonly used in biomarker research studies. These findings highlight that comparison of IL-6 values across COVID-19 research studies requires consideration of the biomarker platform used. Moreover, use of IL-6 as a predictive biomarker for treatment with tocilizumab will require identifying IL-6 thresholds using the same biomarker platform.

**Summary points**

- Plasma IL-6 concentrations were measured on 191 COVID-19 patients using the Roche Elecsys IL-6 assay on the Cobas immunoassay analyzer and the Meso Scale Discovery (MSD) assay.
- Among 191 COVID-19 patients, Roche IL-6 levels had a median (interquartile range) of 62.0 pg/ml (24.7–224.6) and MSD IL-6 levels had 22.0 pg/ml (9.3–74.7).
- The correlation between Roche and MSD was high (Pearson's correlation: r = 0.87; 95% CI: 0.82–0.89).
- Absolute IL-6 levels were consistently higher in samples measured using Roche compared with MSD (bias: 147.2 pg/ml; 95% limits of agreement: -489.5–783.9 pg/ml).
- The optimal IL-6 threshold to predict invasive mechanical ventilation and in-hospital mortality were 3- and 3.4-fold higher in Roche compared with MSD, respectively.
